# Daily eudaimonic well-being as a predictor of daily performance: A dynamic lens

**DOI:** 10.1371/journal.pone.0215564

**Published:** 2019-04-19

**Authors:** Małgorzata W. Kożusznik, José M. Peiró, Aida Soriano

**Affiliations:** 1 Katholieke Universiteit Leuven, Leuven, Belgium; 2 University of Valencia, IDOCAL, Valencia, Spain; 3 Valencian Institute of Economic Research (IVIE), Valencia, Spain; University of Jyvaskyla, FINLAND

## Abstract

Sustaining employees’ well-being and high performance at work is a challenge for organizations in today’s highly competitive environment. This study examines the dynamic reciprocal relationship between the variability in office workers’ eudaimonic well-being (i.e., activity worthwhileness) and their extra-role performance. Eighty-three white-collar employees filled in a diary questionnaire twice a day, once in the morning and once in the afternoon, on four consecutive working days. The results show that eudaimonic well-being displays clear variability in a short time frame. In addition, Bayesian Multilevel Structural Equation Models (MSEMs) reveal a significant positive relationship between the levels of state eudaimonic well-being in the afternoon and the increase in the levels of state extra-role performance from that afternoon to the next morning. Moreover, the overall levels of self-reported state eudaimonic well-being across the diary measurements are significantly and positively related to the overall levels of extra-role performance assessed by the supervisor during the diary measurement. Finally, there is a significant negative relationship between the amount of intra-individual variability in state eudaimonic well-being during the week and the overall levels of self-rated state extra-role performance during the same week. These findings shed light on the dynamic nature of both the eudaimonic component of well-being and performance, highlighting the importance of eudaimonic well-being for extra-role performance and expanding the happy-productive worker thesis. The results suggest that the daily eudaimonic experience of meaning at work should complement the experience of hedonic well-being because it is an important factor in achieving better and more sustainable employee performance on a daily basis.

## 1. Introduction

In organizations, psychologists have often tried to focus on both employees’ performance and well-being in order to achieve sustainable well-being and performance over time [1] and a fair exchange between workers and their organizations. For this reason, the happy-productive worker thesis, which postulates that “happy” workers should have better performance than “less happy” ones [2,3], has been popular and highly researched for over 70 years [4]. However, it has yielded ambiguous and inconclusive results [3,5].

Well-being and performance at work are dynamic phenomena [6,7]. However, most studies on well-being and its consequences have adopted a cross-sectional and static approach [8], investigating their rather stable relationships. Therefore, a dynamic research approach (e.g., diary, Experience Sampling Method, ESM) are needed to capture the changing nature of well-being and performance states [6,9].

Well-being at work can be conceptualized both from a hedonic perspective of pleasure and an experience of positive affect [10] and from an eudaimonic view of an experience of fulfillment and purpose [6], personal growth, a sense of meaning at work [11] and in life [12], and the *worthwhileness* associated with work activities [13,14]. Nevertheless, little is known about how the experience of eudaimonic well-being might affect performance-related processes and the “*the dynamics of eudaimonic well-being at work have remained largely unexplored*” [6].

Performance is a complex construct with several facets, such as task or in-role performance and context or extra-role performance, including discretionary efforts that exceed the job description. The role of the eudaimonic facet of well-being and its relationship with the extra-role dimension of performance is important because, according to Self-Determination Theory (SDT) [15], people who identify with actions carried out at work by accepting or owning them as personally important will intrinsically motivate their behavior at work [15], enhancing their performance, persistence, and creativity [16,17], which are key aspects of citizenship or extra-role performance [18,19]. Specifically, doing what is necessary to fulfill the mission at work, developing one’s identity, self-realization, and doing more than merely fulfilling job tasks all require an eudaimonic focus. It is not possible to be intrinsically motivated if the focus is only on the hedonic aspect of well-being [20]. Therefore, a new challenge emerges to better understand eudaimonic well-being in the work context and expand the current research to include its dynamics, making it possible to understand how to stimulate the sustainability of extra-role performance and well-being.

Furthermore, some authors suggest [21] that the inconsistent evidence about the happy-productive worker thesis may be attributed to the fact that the effect of within-person fluctuations on performance has been neglected. Accordingly, the meaning-making theory [22] suggests that frequent loss of sense of purpose in work activities may have negative effects on employees’ outcomes. Empirical evidence has shown a negative impact of the intra-individual variability (IIV) in hedonic well-being on performance [23,24], and that an episode of a *loss* of sense of purpose in work activities can negatively affect subsequent work performance [25]. However, to our knowledge, no research has studied the dynamic effects of IIV in eudaimonic well-being on extra-role performance.

With this in mind, the aim of this study is to analyze the dynamic nature of eudaimonic well-being and its relationship with extra-role performance. Specifically, this study aims to: 1) shed light on the extent to which state eudaimonic well-being displays short-term fluctuations; 2) uncover the causal dynamic and reciprocal relationship between state eudaimonic well-being and state extra-role performance; and 3) discover whether intra-individual variability in state eudaimonic well-being is discernable by the employee and by others, in that it can be perceived through changes in the overall performance levels (self-rated and assessed by the supervisor).

Achieving these research objectives will allow us to provide novel empirical evidence about the dynamic and complex nature of the relationships between eudaimonic well-being and extra-role performance. From a practical point of view, knowledge about how extra-role performance unfolds over time as a function of eudaimonic well-being, and vice-versa, would make it possible to design intervention strategies to stimulate trajectories of positive change and ensure sustainable performance, producing human and economic benefits for individuals and organizations.

### 1.1. Sustainable performance as a dynamic relationship between well-being and performance

Evolutionary history indicates that displaying generous behaviors and helping others are factors that lead to humans’ success [26,27]. The behavior of employees who “go the extra mile” facilitates fulfilling the organizational mission [28]. Especially now in the rapidly-changing external environment of the current knowledge-based economy, work roles are less clearly defined, and organizations need employees who can easily adopt extra-role emergent behaviors that exceed explicit job requirements in order to help them remain competitive [19]. In work and organizational psychology, researchers have tried to investigate and promote synergies between well-being and employees’ performance in order to achieve the *sustainability* of performance over time. Failing to maintain performance is a threat to contemporary organizations that produces unnecessary costs [29].

Performance sustainability can be viewed as a continuing symbiosis and a dynamic mutually reinforcing relationship with well-being at work that may be considered in its hedonic (e.g., job satisfaction, positive emotions) and eudaimonic (e.g., purpose, personal growth, meaning at work) facets. However, some facets of well-being and performance are especially relevant today. The current economic crisis and difficult times of great employment uncertainty [30] might have made workers realize how much their jobs mean in their lives, making psychological meaningfulness and worthwhileness at work an important issue in the contemporary workplace [31]. Therefore, the study of eudaimonia is as critical in 21^st^ century behavioral science agendas as it was in ancient philosophies [20]. In recent decades, since the emergence and impetus of Positive Psychology [32], more attention has been paid to the study of eudaimonic well-being [33,34], suggesting that it is especially relevant to study its facets related to valuing and fulfilling the mission at work, which help to develop the individual’s identity and self-realization.

We understand performance to be “*a function of a person’s behavior and the degree to which this behavior helps the organization to obtain its goals*” [35,36]. In this regard, extra-role performance refers to certain behaviors that are optional in nature [37] and exceed an employee’s formal job requirements and prescribed tasks [27]. The popular conceptualization of extra-role performance describes it as organizational citizenship behavior, defined as workplace behaviors that are discretionary and not explicitly prescribed, required, or rewarded by an organization. Together, these behaviors support the social and psychological environment in which the central tasks of organizations are accomplished [38], and they promote effective organizational functioning [39]. Specifically, organizational citizenship behavior includes behaviors directed at the organization (e.g., staying extra hours to finish or improve a report), and those directed at individuals (e.g., helping a new employee to find his or her way around) [40,41]. Capturing the changing nature of well-being and performance [6,7] [6,7] requires a dynamic research approach (e.g., using a diary study and ESM), as an alternative to the static cross-sectional approach predominant in the literature [8].

### 1.2. The need to study the dynamics of eudaimonic well-being

In recent decades, ESM and diary studies have blossomed in the organizational research [42] and different studies have paid attention to the relationships between the dynamics of well-being and its outcomes [6]. Nevertheless, the majority of them have only focused on the hedonic aspect of well-being, whereas the consideration of the eudaimonic experience of meaning at work [11] over time [43] has hardly been studied.

Eudaimonic well-being can be assessed as both a trait and a state [44]. In contrast to the trait approach, the state refers to all the experiences that may vary within the same person in response to the changing characteristics of the environment [45,46]. As specified in a literature review by Huta and Waterman [44], *experiences* of state eudaimonic well-being represent a person’s momentary subjective feelings, emotions, and cognitive-affective appraisals (e.g., feeling of meaning). In fact, state experiences stemming from meaningful activity are the basic component in weaving purposeful meaning in the longer term. Thus, to capture these eudaimonic experiences, the recent progress made in subjective well-being measures [47] recognizes the “worthwhileness” associated with work activities as an essential component of eudaimonic well-being at work that is complementary to activities people find “pleasurable” (i.e., hedonic well-being) [13,14]. Indeed, Dolan and colleagues [13] recommend *activity worthwhileness* as a measure of eudaimonic well-being in order to enhance its monitoring. Well-being can fluctuate within weeks, days, or even hours [6], and a useful aspect in describing the dynamics of well-being in work contexts is the concept of IIV [6,48,49], referred to as reasonably short-term, reversible, and rapid changes within subjects [48]. It is reasonable to expect that eudaimonic well-being will present IIV or fluctuations. First, the perception of meaning is essentially a constant fluid process that reflects the current stream of life [50]. According to the meaning-making theory framework [22], situational meaning refers to an ongoing set of processes and outcomes, including the appraised meaning of a particular event, which may be instantaneously determined, but will be subject to continuous revision [51–53]. At work, some activities can feel quite worthwhile some of the time, but quite pointless at other times, provoking feelings of lack of purpose or pointlessness [43]. However, until now, the research in the area of eudaimonic well-being has mainly focused on general individual dispositions (i.e., traits) or overall experiences over longer time periods [54]. They have been measured with questionnaires that capture overall evaluations of purpose in life (or job), whereas state eudaimonic well-being and day-to-day fluctuations in experiences of work activity worthwhileness have remained understudied [6]. Therefore, the first aim of the present study is to shed light on the extent to which state eudaimonic well-being displays short-term fluctuations.

This micro-approach to examining eudaimonic well-being in the time and situation where they occur is beneficial because it allows its proximal outcomes to be investigated with more precision [55]. Therefore, studying both state and trait eudaimonic well-being offers new insights into the psychological mechanisms that explain the variability in employees’ eudaimonia, as well as its relationship with their performance, which would be impossible to obtain if we focused merely on the trait or hedonic facet of well-being [6].

### 1.3. The dynamic relationship between the levels of state eudaimonic well-being and state extra-role performance

Individuals are spending increasingly more time at work, and their work is becoming a greater source of meaning and identity in their lives [56,57]. For a long time, researchers have discussed the experience of psychological meaningfulness at work as an important factor that influences employees’ behavior [58–60]. About half a century ago, Maslow [61] pointed out that individuals who do not believe their work has meaning and purpose will not be able to achieve their full professional potential. Lack of meaning at work can produce disillusion, which, taken to an extreme, can even lead workers to leave their jobs [59].

According to Self-Determination Theory (SDT) [15], organizational contexts have the capacity to foster greater internalization and integration of organizational values, which, in turn, has great significance in increasing their commitment, effort, performance [15], persistence, and creativity [16,17]. Moreover, from the SDT perspective on eudaimonia [20], people who pursue worthwhile goals and values and are mindfully self-regulated are likely to be more socially responsible [20]. A greater internalization of values includes more behavioral effectiveness, greater volitional persistence, and better assimilation of the individual within his or her social group [15], all relevant aspects of extra-role performance.

Specifically, SDT posits that individuals can identify with an action carried out at work if it has intrinsic interest and meaning for them [62] or if they evaluate it and make it congruent with their other values and needs through integration, which will motivate their behavior at work [15]. Thus, employees who support the mission of the organization work toward fulfilling their own mission at work, which may often be manifested in carrying out tasks not prescribed in the job description. By contrast, a person who does not perceive meaning in his/her work will probably alienate him/herself or become ‘disengaged’ from it [63].

Some empirical evidence suggests that meaningfulness is positively associated with internal work motivation [64] and work engagement [65]. Previous research has demonstrated that people who pursue intrinsic goals and values for their own sake and are motivated by the meaning they see in their activities behave in more prosocial ways and show more care, concern, and responsibility in their actions, thus benefiting other people (e.g., colleagues at work) [20]. Moreover, based on the results of McHoskey [66], people guided by intrinsic goals are less likely to display Machiavellian behavior and more likely to have social interests.

We might expect a general eudaimonic experience of meaning at work to unfold in longer cycles because it involves self-realization and the perception of purpose at work, suggesting a long-term perspective. However, it is reasonable to expect that fluctuations in worthwhileness associated with the meaning of specific activities one performs at work on a daily basis may unfold in short-term cycles or on a more micro level. Indeed, some empirical evidence suggests that this relationship exists. For example, Niessen and colleagues [67] showed that on days when employees perceived increased meaning at work, they also reported behaving in a more exploratory way (i.e., carrying out more information searches), compared to days when they perceived that their work had less meaning for them. In spite of this, little is known about how the experience of eudaimonic well-being might affect performance-related processes, especially those concerning extra-role performance [6]. An additional issue in disentangling this relationship is the operationalization of performance based on the source of its appraisal. Two main sources are considered in the literature: self-appraisal and supervisor appraisal. Each of them provides relevant information, and meta-analyses indicate that their correlation is rather modest (*p* = .35) [68]. Self-appraisal reports on how employees perceive the relationship between their subjective meaning and their extra-role behaviors, whereas supervisors’ appraisal, when related to employees’ reports of changes in meaning, indicates that the effect of employees’ perceptions of work worthwhileness on performance is perceived by others.

Based on the above, we can expect that, when people experience greater state eudaimonic well-being at work, they will display better state extra-role performance. Therefore, we formulate the following hypotheses:

*Hypothesis 1*: The levels of self-rated state eudaimonic well-being at one measurement point will be positively related to the change in self-rated state extra-role performance from that point to the next measurement point (i.e., from the morning to the afternoon and from the afternoon to the end of the next morning), above and beyond the impact of the levels of self-rated state hedonic well-being.
*Hypothesis 2*: The overall levels of self-rated state eudaimonic well-being across the diary measurement will be positively related to the overall levels of extra-role performance assessed by the supervisor, above and beyond the impact of the levels of self-rated state hedonic well-being.

### 1.4. Possible reciprocal relationships

Most organizational research has studied performance as an outcome variable. However, this approach only paints a partial picture because work can help employees to develop their identity, making them eudaimonically happier and more developed [15]. Several theories suggest this inverse relationship between well-being and performance, and there is evidence that within-person dynamics in extra-role work performance can explain within-person dynamics in well-being indicators [6].

First, Rosso, Dekas, and Wrzesniewski [11], in their attempt to synthesize the literature on sources of meaning in work, revealed that the extent to which one perceives that he or she is making a contribution or a significant impact on others is a pathway to increased meaning in work. Thus, experiencing work as meaningful may arise from performing work that contributes to the common good [11].

Second, according to organismic integration theory (OIT) [62], a sub-theory of SDT, employees’ experiences of satisfaction of the need for competence, autonomy, and relatedness in the workplace promote internalization and integration of the activities carried out at work [15] as holding personal meaning, and predict their well-being at work [69], even at a daily level [70]. Behaviors characteristic of high-quality extra-role performance manifested as greater competence, being involved in volitional and social activities, making community contributions, and generally altruistic or generative acts should satisfy all three needs fairly directly [20]. This can also be explained by classic humanistic psychology and motivation theories that propose that people experience meaningfulness when they feel like they make a difference [58].

Finally, the Job Characteristics Model (JCM) [60] suggests that greater perceived task significance at work by employees is produced by greater frequency, physical proximity, duration, depth, and breadth of contact with beneficiaries of these activities [71]. Accordingly, we understand that when a person perceives his/her state extra-role performance to be excellent, this perception can boost his/her state eudaimonic well-being because s/he internalizes the values and considers the activities at work to be more worthwhile.

There is also empirical evidence supporting the opposite relationship between well-being and performance. The majority of it considers the hedonic facet of well-being. For example, longitudinal studies have shown that self-rated performance is a predictor of increased dedication and decreased emotional exhaustion over time [72]. Moreover, studies indicate that performance [73,74] and the experience of making progress toward one’s goals at work [75–77] are predictors of positive affective states. From the eudaimonic perspective, Huta and Ryan [78] found that these eudaimonic activities (which involve e.g., relationships, community goals, mindful and aware acting, behaving in autonomous and volitional ways, and behaving in ways that satisfy basic psychological needs for competence, relatedness, and autonomy) are positively related to several measures of meaning in life. These motivational concepts are relevant to extra-role performance because they refer to behaviors that exceed what is included in a job description and broader, mindful, aware, and fulfilling community-related goals and values that are relevant to the organization. Moreover, there is some evidence about causal relationships from experimental studies, where scholars show that higher levels of task meaning are accompanied by higher output levels [25,79,80], such as more effort [80] and an increase in quantity and quality [79].

A few studies show the possible dynamic relationship between the levels of performance and eudaimonic well-being. For example, on days when employees focused strongly on their tasks at work, they presented higher levels of vitality and learning than on days when their task focus was weak [67]. Another study shows that personal initiative predicts an increase in work engagement over time [81].

Taking all of the above into account, we suggest that people who evaluate themselves as having high extra-role performance at work will display greater state eudaimonic well-being, in terms of considering their activities at work to be more worthwhile and meaningful. Therefore, we formulate the following hypothesis:

*Hypothesis 3*: The levels of self-rated state extra-role performance at one measurement point will be positively related to the change in self-rated state eudaimonic well-being from that point to the next measurement point (i.e., from the morning to the afternoon and from the afternoon to the end of the next morning).

### 1.5. The impact of intra-individual variability in eudaimonic well-being on extra-role performance

Some researchers suggest that, in addition to the impact of the levels of meaning at work, the *fluctuations* in meaning at work can have an important deleterious impact on work performance. First, following Kahn’s [58] psychological conditions framework, people may vary in their personal engagement in their performance at work based on their perceptions of the meaningfulness of the situations (e.g., a feeling of worthwhileness) and the safety they perceive in them. This safety is associated with elements of social systems that create more or less nonthreatening, predictable, and consistent social situations in which their personal engagement would not suffer [58]. Therefore, the conditions for employees’ engagement should be present when carrying out worthwhile activities at work, which means they should remain consistent (e.g., such as showing low fluctuations) over time.

Second, according to meaning-making theory [22], frequent loss of a sense of purpose in work activities may be a powerful generator of distress [82], and it can have negative outcomes in terms of performance [25]. These considerations agree with the view of variability as non-adaptive in terms of its negative short-run correlates and outcomes in general. Specifically, in the experiential domains, high levels of short-term within-person fluctuations can reflect a lack of robustness or frailty of the system [23]. Empirical findings support this view. For example, greater IIV in hedonic well-being (i.e., negative affect) is negatively related to cognitive performance [24]. From the eudaimonic perspective, recent experimental research shows that loss of meaning in a previous task leads to a considerable drop in agents’ effort and performance levels, even on an unrelated follow-up task [25].

A person’s fluctuations in his/her central tendency on some variable can be operationalized as IIV [83]. IIV refers to fluctuations, inconsistency, instability, or oscillations that appear on micro-time scales (e.g., minutes, hours, days, weeks) [49]. The most frequent [23] operationalization of the amount of variability in a construct or the univariate net variability characteristic is the intra-individual standard deviation (i*SD*), which is easy to calculate, has immediate face validity and has been used to reliably measure intra-individual variability in hedonic well-being (i.e., affect) [84]. Following Ram and Gestorf [49], in our case, an i*SD* calculated for the distribution of scores obtained across repeated measurements of the state eudaimonic well-being of a single individual would describe the extent to which his or her scores on this variable tend to fluctuate in time around the mean score. Accordingly, a large i*SD* would indicate that the individual had a wide range of perceptions of state eudaimonic well-being worthwhileness (e.g., high fluctuation), whereas a small i*SD* would indicate a narrow range of perceptions (e.g., low fluctuation).

Analyzing IIV by taking into account the dynamics in variables during subsequent days or weeks is a promising development in the field of analysis [7]. Moreover, investigating short-term fluctuations in well-being may further explain inconsistent evidence gathered in the framework of the happy-productive worker thesis [2,21].

Taking all of this into account, we propose that high variability in state eudaimonic well-being (indicating frequent gains and losses in the sense of activity worthwhileness) could have negative effects on employees’ outcomes in terms of their deteriorated state extra-role performance. Therefore, we formulate the following hypothesis:

*Hypothesis 4*: Intra-individual variability in self-rated state eudaimonic well-being will be negatively related to overall extra-role performance assessed by the supervisor across the studied period, above and beyond the impact of the intra-individual variability in self-rated state hedonic well-being.
*Hypothesis 5*: Intra-individual variability in self-rated state eudaimonic well-being will be negatively related to the overall self-rated state extra-role performance across the studied period, above and beyond the impact of the intra-individual variability in self-rated state hedonic well-being.

### 1.6. Supervisor ratings

An additional feature of the present study is that it combines employees’ ratings of their own state extra-role performance with the examination of overall levels of employees’ extra-role performance during the measurement period as assessed by their direct supervisors. We consider it necessary to complement employees’ ratings of their state extra-role performance with the supervisor’s evaluation of their general levels of extra-role performance because both sources of appraisal provide relevant information and can help to overcome the biases of the other assessment source. Furthermore, it is relevant to investigate whether one source of appraisal confirms the relationships found with the other one.

Given that it is the employees who experience the change in the meaning given to their work activities and its effect on their intrinsic motivation and engagement, they will notice the oscillations in their performance more. Thus, including employees’ self-ratings may help to more precisely describe the relationship between their well-being and performance.

In turn, including supervisor ratings could help to overcome the problem of employees’ leniency or self-deception in self-ratings, which has been shown to be especially pronounced in the case of general or trait judgments of performance [85], possibly because they address not only past behavior, but also respondents’ expectations of current and future behavior [86]. Therefore, by using self-reports along with supervisor evaluations of employees’ overall levels of extra-role performance, we ensure that we are using two complementary evaluations, which can give us a unique and a richer perspective on performance from different sources.

### 1.7. The adequate temporal lens

Finally, in order to carry out a study of the dynamics of the relationship between state eudaimonic well-being and state extra-role performance and see how these phenomena unfold over time in people’s work, we need to select an adequate *temporal lens* [87]. According to Barker [88], the continuous flow of daily behavior could be segmented into natural units or *behavior episodes* that can vary every day and within each day. Likewise, based on the episodic process model of affect and performance [42], performance can follow similar patterns, showing short-term within-person variability and fluctuating from day to day (circadian rhythms are an important phenomenon here) or even within a person during a day. Indeed, performance trajectories generally follow a pattern that may be repeated throughout the day and the week [7].

Similarly, the daily level of analysis is also relevant for studying the dynamics of well-being because well-being fluctuates within shorter periods of time (e.g., weeks, days, or even hours) [6]. Empirical evidence agrees with these theoretical considerations and suggests that what happens at work at a specific time or on a specific day is the key to understanding within-person fluctuations in employee well-being [21]. Importantly, Sonnentag [6] pointed out that research should be based on more fine-grained measurements that would include more than one assessment per day in order to gain more insight into the dynamics of the underlying processes.

Taking into account the episodic approach, we consider that the time frame of a work week with measurements twice a day (morning and afternoon) on four consecutive days is a reasonable temporal lens from which to study the dynamics of state eudaimonic well-being and state extra-role performance. This fine-grained assessment with more than one measurement per day can help to clarify whether the state variables on the morning of the same day will affect their outcomes during the afternoon of the same day in a similar way as the state variables in the afternoon would affect their outcomes during the next morning. In addition, this approach will help to explain the relationship between the intra-individual variability over a week and the overall levels of the outcome variables. These complex questions about the dynamics of state eudaimonic well-being and state extra-role performance may open up Pandora’s box because many issues still have to be clarified.

## 2. Method

### 2.1. Sample

In this study, we used a multilevel diary design applied to a sample of 83 office workers and their direct supervisors (68.7% women; mean age 39.67, *SD* = 8.85). In order to maximize the representativeness of the sample, we approached white-collar office workers in five organizations in Spain from different sectors: higher education, public sector (1); furniture industry and banking, both from the private sector (2); and R&D and professional services, mixed sector (2), which are important sectors of activity in the Spanish context and frequently use offices as work environment for different jobs.

The process of selecting the participants in the organizations was not random. Specifically, we wanted to ensure that we included employees and their direct supervisors working in all the office types described in the widely established office typology proposed by Neufert [89] with regard to spatial requirements, which divides offices into: cellular offices, group offices, and open-plan offices. Thus, our aim was to increase the diversity of office work environment and, consequently, the type of activities in office jobs. Based on the findings from simulation studies about power in multilevel models [90], we wanted to have a sample of at least 50 participants at level 2 (participants) in order to ensure the accuracy of the estimates.

The majority of the employees in each organization work full-time (69%) on a permanent contractual basis (79%). Even though the companies belong to different sectors, the office work is, to a great extent, transversal across all these sectors, as it includes functions such as bookkeeping, purchase management, administration, and other typical office tasks that are fairly similar in different sectors. Moreover, the work settings (often the offices) tend to be similar across the organizations, compared to other types of activities directly related to specific sectors. Finally, the results of Box’s M statistic (*p* = .33) indicate that the variance-covariance matrix of the variables of interest can be assumed to be equal in the five organizations.

The majority of the employees were married/living with a partner (72.3%), whereas one out of 4 were single (26.5%), and only a small proportion were separated/divorced (1.2%). With regard to the highest education level reached, the majority of the sample hold MA/Msc university degrees (50.6%), 26.5% were university graduates, 13.3% had finished occupational training, 8.4% had PhDs, and 1.2% had completed compulsory education (primary or secondary). The sample included managers (4.8%), highly qualified professionals (32.5%), technicians (25.3%), clerks (32.5%), and others (4.8%). The number of working hours per week ranged from 20 to 45 (*M* = 31.31, *SD* = 6.35). The commute time from home to work ranged from 5 minutes to 2 hours (*M* = 20 minutes, *SD* = 17 minutes). Finally, the distribution of the net salary after taxes was the following: less than 600€ (7.2%), between 600€ and 1000€ (4.8%), between 1000€ and 1499€ (41.0%), between 1500€ and 1999€ (27.7%), between 2000€ and 3000€ (18.1%), and more than 3000€ (1.2%).

In this study, we collected the data from the office workers over the course of a work week, with measures on four consecutive days, twice a day. Because some of the respondents were away from the office during part of the workday, we failed to collect data at 61 time points. Therefore, we obtained 603 data collection points.

### 2.2. Procedure

Self-reported state hedonic well-being, state eudaimonic well-being, and state extra-role performance were measured twice a day (once at the end of the morning and once at the end of the afternoon) on four consecutive days using a diary questionnaire. Each employee has given his/her written consent to participate in this study. We intended to collect data from each of the employees in their offices at the same time in the morning and in the evening; however, due to the limited availability of some participants at their workstations, there were some differences in the data collection times. Each employee’s baseline extra-role performance was evaluated by his/her direct supervisor at the end of the diary data collection week using a short questionnaire. Each supervisor evaluated between 1 and 19 of their own subordinates (an average of 5.13 employees evaluated per one supervisor). Participation in the study was voluntary, and the data were treated in an anonymous and confidential way. The study was approved by the institutional Ethics Committee.

### 2.3. Measures

#### 2.3.1. State well-being and state performance

The *state hedonic well-being* scale measures the extent to which a person experiences positive emotions at work. It was measured using a 3-item positive emotions scale (e.g., “Happy”) [14], based on the Day Reconstruction Method by Kahneman, Krueger, Schkade, Schwartz and Stone [91]. The respondents were asked to indicate how they had been feeling at work in the past couple of hours, using a response scale ranging from 1 (not at all) to 7 (very much), where 1 means that the person was not experiencing any feeling at all, and 7 means that the person was experiencing a strong feeling. The average Cronbach’s alpha for state hedonic well-being was .73, and it ranged between .68 and .77 across the eight measurement points. Cronbach’s alphas for all diary variables are mean internal consistencies averaged across all the measurement points.

*State eudaimonic well-being* refers to an individual’s perception that the activities s/he carried out previously were worthwhile and useful to other people, had greater meaning, and served a higher purpose. It is measured with a 3-item scale [14]. The respondents were asked to indicate whether they felt the activities they had been doing in the past couple of hours were “…worthwhile and meaningful” (sample item). The response scale ranged from 1 (not at all) to 7 (very much). The average Cronbach’s alpha across the eight measurement points for the eudaimonic well-being scale was .79, ranging between .75 and .82.

Finally, *state extra-role performance* refers to behaviors that are not directly related to the tasks included in the job description and are optional in nature, such as helping others or doing more than an employee’s formal job requirements. It is measured using 3 items adapted from Goodman and Svyantek’s [92] scale to measure contextual performance. We wanted to keep the measure short in order to minimize response biases caused by boredom or fatigue [93]. Therefore, we retained three items because it has been suggested that this is the minimum number of items needed to obtain adequate internal consistency reliabilities [94,95]. Exploratory and Confirmatory Factor Analyses showed adequate factor loadings of the items. The respondents were asked to indicate on a scale ranging from 1 (completely disagree) to 7 (completely agree) the extent to which they agreed with a series of statements about the work they had been doing in the past couple of hours. A sample item was “(In the past couple of hours…) …I have been helping other colleagues who have been absent”. The average Cronbach’s alpha across the eight measurement points for the extra-role performance scale was .74, ranging between .69 and .76.

#### 2.3.2. Variability in state hedonic well-being

Intra-individual variability in state hedonic well-being for each person was quantified by the intra-individual standard deviation (i*SD*) in state hedonic well-being, which consists of a mean standard deviation in individual scores across the eight measurement points, measured with the 3-item scale (e.g., “Happy”) [14], based on the Day Reconstruction Method by Kahneman and colleagues [91] described above.

#### 2.3.3. Variability in state eudaimonic well-being

Intra-individual variability in state eudaimonic well-being for each person was quantified by the i*SD* in state eudaimonic well-being, which consists of a mean standard deviation in individual scores across the eight measurement points, measured with the 3-item scale [14] described previously.

#### 2.3.4. Overall performance

Overall levels of extra-role performance were measured using a 3-item scale analogical to the one described in the previous section [92], but adapted to capture the employee’s overall extra-role performance. In this case, the employee’s direct supervisor was asked to indicate his/her level of agreement with each statement, such as “(The employee at work…) …helped other employees with their work when they had been absent”, using a response scale ranging from 1 (totally disagree) to 7 (totally agree). The Cronbach’s *a* for the scale was .86.

### 2.4. Analyses

In the present study, we have data from each person at two levels: at the person level (Level 2) and at the day level (Level 1), with day-level data nested within persons. Variability in state eudaimonic well-being and the overall levels of extra-role performance assessed by the supervisor constituted the Level 2 data. Day-level measures of state eudaimonic well-being and state extra-role performance (both measured in the morning and in the afternoon on four consecutive days) constituted the Level 1 data.

We centered person-level (Level-2) predictors around the grand mean and day-level (Level-1) predictors around the respective person mean. We decided to center variables at Level 1 around the respective person mean in order to eliminate between-person variance and attribute effects of Level 1 variables to within-person effects, ruling out interpretations based on between-person differences [96]. Thus, we were not interested in whether the absolute level of state eudaimonic well-being in the morning/afternoon is related to the increase in absolute state extra-role performance (during the same day or from the evening of the previous day to the next morning). Instead, we were interested in whether a higher or lower state of eudaimonic well-being in the morning within a person (i.e., compared to the respective mean of this individual) is related to the increase in state extra-role performance during the same day. Similarly, we were interested in finding out whether a higher or lower state of eudaimonic well-being in the afternoon within a person is related to the increase or decrease in state extra-role performance from the afternoon of the previous day to the next morning [96].

In order to address the first aim of this study, namely, to shed light on the extent to which state eudaimonic well-being displays short-term fluctuations, we investigated the relative amount of variance in the study variables between- and within-persons by inspecting the intraclass correlation coefficient Type I (ICC1) [97]. Within the context of a nested data structure, the ICC1 makes it possible to determine the percentage of variability in state eudaimonic well-being within and between individuals across the eight measurement points (twice a day on four consecutive days) during a work week. Specifically, as the ICC1 is defined as the proportion of total variance that can be explained by group membership [98], it reflects the amount of between-individual variability for a variable of interest relative to the total variability (the sum of between-individual and within-individual variability) [99]. Thus, large ICC1 values would reflect large differences in state eudaimonic well-being between individuals, but small differences in state eudaimonic well-being within individuals. A large ICC1 would reflect stability in eudaimonic well-being, whereas a small ICC1 (e.g., below .10) [100] might suggest that eudaimonic well-being varies more from one assessment to another.

In order to address Hypotheses 1–3 and 5, we used first-order Multilevel Structural Equation Modeling (MSEM) with Bayesian estimations. In the case of Hypotheses 1 and 3, the outcome variables were calculated by saving the unstandardized residual scores from the regression analyses, using the state variable measured in the previous time point as a predictor and the state variable measured in the next time point as the dependent variable. The outcome variable in Hypothesis 2 was an overall score for the extra-role performance of each employee, assessed by his/her direct supervisor. In the case of Hypothesis 5, the outcome variable was the overall self-rated state extra-role performance, calculated using the eight scores for self-rated extra-role performance during the same week, nested within persons.

As this study describes the first attempt to study the dynamic relationship between state eudaimonic well-being and state extra-role performance (and vice-versa), we have specified an uninformative prior distribution reflecting no prior knowledge, as recommended by Van de Schoot, Kaplan, Denissen, Asendorpf, Neyer and Van Aken [101]. Therefore, in our analyses, we have chosen the default uninformative priors provided by Mplus; for the specification of the defaults, see Asparouhov and Muthén [102]. For all loadings and intercepts, the prior is uniform on the (−∞, ∞) interval [102]. Following the suggestion of Asparouhov and Muthén [102], we used a large number of MCMC iterations (100,000) and investigated the stability of the parameter values across iterations. Thus, as Hox, van de Schoot and Matthijsse [103] recommend, we set stricter criteria for convergence, reducing the bias in the residual variances at the person level, while greatly increasing the computation time. We requested multiple chains of the Gibbs sampler by using chains = 4 [101].

In the Bayesian context, model fit refers to assessing the predictive accuracy of a model, and it is called posterior predictive checking [104]. Posterior predictive checking serves to evaluate the specification quality of the model from the perspective of predictive accuracy [101]. In order to assess the fit of a Bayes model, the Posterior Predictive P-value (PPP) is offered. It is defined as the proportion of chi-square values obtained in the simulated data that exceed that of the actual data. PPP values around .50 indicate an excellent model fit [105]. Mplus provides the Potential Scale Reduction (PSR) convergence criteria [106], where the PSR ≤ 1.1 indicates good convergence [104].

Benefits of Bayesian statistics are recognized in the literature [107,108]. Specifically, these advantages are that: a) Bayesian estimation works well with smaller sample sizes [103]; b) it solves the problem of negative variance estimates or correlations larger than 1, as the estimates are always proper due to the correct probability distribution [101]; c) it can deal with asymmetric distributions [103], making it possible to provide more accurate results [101], even with complex models [103]; and d) it offers Posterior Probability Intervals (PPIs), also called credibility intervals, which refer to the 95% probability that in the population a certain parameter lies between two numbers [101]. These issues are relevant for our complex multilevel models, which deal with diary data from 83 persons and several variables that have not demonstrated normal univariate distribution by showing asymmetry and kurtosis values beyond the limit of ±2 [109–111].

In order to address Hypothesis 4, we calculated the IIV (quantified by the intra-individual standard deviation, i*SD*) in state eudaimonic well-being for each person. Next, using this data as the predictor and the general performance levels evaluated by the supervisor as the outcome, we ran linear regressions in SPSS. We controlled for the impact of intra-individual variability in state hedonic well-being.

Because repeated-measures data for the latent variables have to retain their meanings across all the data collection points [112], prior to modeling change, we tested the longitudinal factorial invariance [113], by conducting Differential Item Functioning (DIF) analysis of the scales used in this study. DIF analysis consists of a comparison of models with different constraints: 1) Structural equivalence; 2) Factor loading invariance; 3) Factor loading and intercept invariance; and 4) Factor loading, intercept, and error invariance. Following Chan [114] and Meredith and Horn [115], for the invariance assumption to be supported, at least factor loading invariance should be ensured on repeated occasions. In order to assess the model fit, we examined the RMSEA (root mean square error approximation), CFI (comparative fit index), TLI (Tucker-Lewis index), and SRMR (root mean square residual) goodness of fit statistics. We considered that an acceptable fit exists when a model fulfills the following criteria: RMSEA ≤ .08, CFI ≥ .90, TLI ≥ .90, SRMR ≤ .10 [116].

## 3. Results

### 3.1. Preliminary results

In the first place, DIF analyses showed that the model with factor loading and intercept invariance obtained the best fit (χ^2^ = 457.99, *df* = 294, *p* < .001, χ^2^/*df* = 1.56, RMSEA = .086 [.070; .101], CFI = .915, TLI = .917, SRMR = .106), indicating fit on the threshold of acceptance for a model with strong factorial invariance and providing support for the assumption that the latent variables used in our study retain the same meaning on all eight repeated occasions.

Means, standard deviations, and correlations between study variables are displayed in S1 Table. In order to shed light on the extent to which state eudaimonic well-being displays short-term fluctuations, we inspected the relative amount of variance in this variable between and within persons by inspecting the ICC1. State eudaimonic well-being had a mean ICC1 for its items of .70, indicating that 70% of the variance in state eudaimonic well-being was between-person variation, whereas 30% lied within persons. These results point out that state eudaimonic well-being displayed a considerable amount of short-term fluctuation.

In the next step, we investigated the dynamic relationships between daily state eudaimonic well-being and state extra-role performance. We took into consideration the different parts of the day (i.e., morning and afternoon). Specifically, we analyzed whether the levels of state eudaimonic well-being in the morning predicted the change in state extra-role performance from morning to afternoon on the same day. We also analyzed whether the levels of state eudaimonic well-being in the afternoon predicted the change in state extra-role performance at the end of the next morning. We also tested the inverse relationship between our variables of interest. The results of all the Bayesian MSEMs are available in Tables 1 and 2. All the models obtained good fit and convergence (*PPP*s > .05 and *PSR*s < 1.1) [101,106]. The results showed a significant positive relationship between the levels of state eudaimonic well-being in the afternoon and the increase in the levels of state extra-role performance the next morning (*PPP* = .49. *IC* [*LL* = -8.34; *UL* = 8.49]. *Est*. = .30. *IC* [*LL* = 0.05; *UL* = 0.56]). These results partially supported Hypothesis 1.

**Table 1:** Multilevel Structural Equation Models predicting state extra-role performance from state eudaimonic well-being.

	Same-day relationships				Previous day-next day relationships			
	Est.	*post*. *SD*	*LLCI*	*ULCI*	*Est*.	*post*. *SD*	*LLCI*	*ULCI*
Within-level								
Intercept	.00	.05	-.09	.09	.00	.05	-.10	.10
State Hedonic WB	-.15	.08	-.31	.02	.22	.12	-.02	.46
State Eudaimonic WB	.02	.08	-.14	.18	.30	.13	.05	.56
Residual variance	.55	.05	.47	.66	.33	.04	.26	.42
*PPP [LLCI; ULCI]*	.50		-8.34	8.37	.49		-8.34	8.49
*PSR*	1.00				1.00			

***Note***. *n* = 80–82 at the person level; *post*. *SD* = posterior Standard Deviation; Average observations per person = 3.57 for same-day relationships and 2.57 for previous day-next day relationships.

**Table 2:** Multilevel Structural Equation Models predicting state eudaimonic well-being from state extra-role performance.

	Same-day relationships				Previous day-next day relationships			
	*Est*.	*post*. *SD*	*LLCI*	*ULCI*	*Est*.	*post*. *SD*	*LLCI*	*ULCI*
Within-level								
Intercept	.00	.03	-.06	.06	.00	.04	-.08	.08
State Extra-Role Performance	-.06	.04	-.14	.02	.09	.08	-.08	.25
Residual Variance	.25	.02	.21	.29	.26	.03	.20	.32
*PPP [LLCI; ULCI]*	.50		-7.20	7.08	.50		-7.21	7.29
*PSR*	1.00				1.00			

***Note***. *n =* 82 at the person level; *Post*. *SD* = posterior Standard Deviation; Average observations per person = 3.57 for same-day relationships and 2.60 for previous day-next day relationships.

In addition, the results of all the Bayesian MSEMs showed a significant positive relationship between the overall levels of self-reported state eudaimonic well-being across the diary measurements and the overall levels of extra-role performance assessed by the supervisor (*PPP* = .02, *PSR* = 1.00, *Est*. = .71, *CI* [*LL* = 0.49; *UL* = 0.93]), when controlling for the overall levels of self-reported state hedonic well-being across the diary measurements. The models obtained a reasonable model-data fit (*PPP* value > .01) [105] and good convergence [101,106]. These results provided support for Hypothesis 2. Because we did not find significant relationships in the other models (ICs including zero), Hypothesis 3 was not supported.

The analysis of linear regressions, where overall extra-role performance assessed by the supervisor was regressed on the IIV in state eudaimonic well-being, controlling for the IIV in state hedonic well-being, showed that the IIV in state eudaimonic well-being is not related to overall levels of extra-role performance assessed by the supervisor (*F* = 0.24, *p* = .79). These results did not support Hypothesis 4.

To test the relationship between the IIV in state eudaimonic well-being and self-rated state extra-role performance, we ran two MSEMs with Bayesian estimation. The results showed that there was a significant negative relationship between the IIV in state eudaimonic well-being during the week and the mean levels of self-rated state extra-role performance during the same week (*PPP* = .27, *CI* [*LL* = -9.47; *UL* = 17.70], *PSR* = 1.00, *Est*. -1.45, *CI* [*LL* = 2.25; *UL* = -0.52]), when controlling for the IIV in hedonic well-being, thus supporting Hypothesis 5 (see Table 3).

**Table 3:** Multilevel Structural Equation Models predicting mean daily levels of state extra-role performance from intra-individual variability in state eudaimonic well-being.

	*Est*.	*post*. *SD*	*LLCI*	*ULCI*
Between-level				
Intercept	-.12	.16	-.43	.20
Variability in State Hedonic WB	-0.06	.43	-0.92	0.78
Variability in State Eudaimonic WB	-1.45	.44	-2.25	-0.52
Residual variance	1.89	.36	1.34	2.74
*PPP [LLCI; ULCI]*	.27		-9.47	17.70
*PSR*	1.00			

***Note***. *n* = 83 at the person level; *post*. *SD* = posterior Standard Deviation; Average observations per person = 7.61.

## 4. Discussion

The present study aimed to analyze the dynamic nature of eudaimonic well-being and its relationship with extra-role performance. Specifically, the purpose of this study was to: 1) shed light on the extent to which state eudaimonic well-being displays short-term fluctuations; 2) uncover the causal dynamic and reciprocal relationship between state eudaimonic well-being at one measurement point and the change in state extra-role performance from this measurement point to the next; and 3) discover whether intra-individual variability in state eudaimonic well-being is discernable by the employee and by others, in that it can be perceived through changes in the overall performance levels (self-rated and assessed by the supervisor).

The results show that eudaimonic well-being reveals a considerable amount of short-term fluctuation. Specifically, 30% of the variance in state eudaimonic well-being is found within persons. These results resonate with the dynamic perspective of well-being [6] and with the meaning-making theory framework [22], which emphasizes situational meaning as an ongoing set of processes and outcomes involving appraisals of the meanings of events or occurrences that are subject to constant revision [51–53]. They also reflect the fluid nature of the perception of meaning that represents the current stream of life [50].

Moreover, the results showed a significant positive relationship between the levels of state eudaimonic well-being in the afternoon and the change in the levels of state extra-role performance the next morning, yielding partial support for Hypothesis 1. We also found support for Hypothesis 2, which stated that the overall levels of self-reported state eudaimonic well-being across the diary measurements would be positively related to the overall levels of extra-role performance assessed by the supervisor. These results agree with researchers who find the experience of psychological meaningfulness at work to be an important factor that can impact employees’ behavior [58,59] and allow them to release their professional potential [61]. They coincide with SDT [15] and the SDT perspective on eudaimonia [20], suggesting that employees whose values are aligned with those of the organizations, who consider their work activities to be full of meaning, and who pursue worthwhile goals will show enhanced performance [15] and greater volitional persistence [16,17].

The results obtained are consistent with the results reported by Niessen and colleagues [67], who showed that, on days when employees perceived increased meaning at work, they also reported higher performance than on days when they perceived their work to be less meaningful for them. The findings also agree with research suggesting that meaningfulness is positively associated with internal work motivation [64], work engagement [65], and behavior that favors social interests [20,66]. Interestingly, the significant positive relationship appears only between the levels of state eudaimonic well-being in the afternoon and the increase in the levels of state extra-role performance the next morning, and not between these variables during the same day. This result can indicate that some mediator variables may be involved in this relationship. For example, it could be related to within-day [117] and after-work-time recovery [118] issues. Indeed, some authors showed that eudaimonic well-being reduces sleep problems [119], which contributes to better recovery [118] and may finally lead to better work performance the next day. This possible mediation relationship should be studied in future research.

Furthermore, we did not find support for Hypothesis 3, which stated that the levels of state extra-role performance at one measurement point would be positively related to the change in state eudaimonic well-being from that point to the next measurement point (i.e., from the morning to the afternoon and from the afternoon to the end of the next morning). The results highlight that there is no reciprocal relationship between extra-role performance and eudaimonic well-being. This means that high daily levels of state extra-role performance do not increase the levels of eudaimonic well-being. These results do not agree with theory and research suggesting that engaging in meaningful work may promote eudaimonic well-being. Namely, they do not agree with the organismic integration theory (OIT) [62], which states that behaviors characteristic of high-quality extra-role performance (i.e., being competent in volitional and social activities, community contributions, and altruistic or generative acts) lead to internalization and integration of the work activities as personally important [15]. These results are not consistent either with research that shows evidence for the inverse [25,79,80] and dynamic [67] relationship between performance and meaning at work. The failure to identify reciprocal results could be due to the fact that, in the present study, we investigated only one aspect of contextual performance (i.e., extra-role performance). Future investigations should study whether other types of performance (e.g., creative performance) contribute significantly to increasing the levels of eudaimonic well-being, taking into account that these types of performance may induce a sense of self-realization to a greater extent.

Finally, the results show that there is a significant negative relationship between the IIV in state eudaimonic well-being during the week and the mean levels of self-rated state extra-role performance during the same week, supporting Hypothesis 5. In other words, people who fluctuate more in their eudaimonic well-being (e.g., by gaining and losing the perception of meaning in tasks) report an overall worse extra-role performance during the work week. By contrast, our data do not support Hypothesis 4, which stated that the IIV in state eudaimonic well-being would be negatively related to overall extra-role performance assessed by the supervisor across the diary measurements.

These results coincide with Kahn’s [58] psychological conditions framework and the meaning-making theory [22], which suggest that *fluctuations* in meaning at work can have an important deleterious impact on work performance. Specifically, these results agree with Kahn’s [58] psychological conditions framework, which points out that individuals’ perceptions of nonthreatening, predictable, and consistent social situations reflected in low fluctuations in activity worthwhileness are predictors of the degree of personal engagement that motivates their performance at work [58]. Furthermore, the results support other studies showing that frequent loss of sense of purpose in work activities can have negative outcomes in terms of performance [25]. In sum, the results show that variability in eudaimonic well-being can be non-adaptive because it has negative short-term outcomes, pointing out the *lack of robustness* or *frailty* of the system reflected in high levels of short-term within-person fluctuations [23].

Our results show that only self-reported extra-role performance is related to eudaimonic well-being, and in the present sample we were unable to show a significant relationship between well-being and performance evaluated by the supervisor. These results suggest that the time frame considered in our study using fine grained measures (morning and afternoon) over four days is effective for the employee awareness well-being fluctuation, but it is probably not the best time frame for supervisor perceptions. The supervisor probably needs longer periods (than just four days) and wider cycles over these periods to perceive changes in performance related to oscillations in eudaimonic well-being. It is possible that in the present study we were able to capture the short-term oscillations in eudaimonic well-being and extra-role performance because the employees are able to notice these oscillations and more precisely report on the relationship between their own well-being and performance. In any case, the fact that the results based on the data from two different informants do not coincide in our study suggests that more research is needed to clarify the dynamic relationship between the levels of state eudaimonic well-being and state extra-role performance, in order to obtain relevant information from both evaluation sources.

### 4.1. Limitations, contributions, and implications

Some limitations warrant a cautious interpretation of the results of this study. First, the measurement of eudaimonic well-being was limited to activity worthwhileness. Future investigations should take into account other components of eudaimonic well-being, such as self-realization or purpose, although these types of eudaimonic well-being facets might require a longer time frame because their fluctuations might take longer to detect than fluctuations in activity worthwhileness. In any case, it is important for future research to analyze these fluctuations in states during short periods of time or on a micro scale because these important alterations in well-being in the short-term could lead to important deteriorations in performance or occasional errors that might affect organizational outcomes or employees’ safety in certain jobs. Second, as mentioned above, the present study focused on analyzing extra-role performance. Future investigations should consider including other types of contextual performance, such as creative performance, in the analysis of the co-fluctuations in state eudaimonic well-being and state performance. Third, although we intended to collect data at the same time in the morning and in the evening, there were some slight differences in the data collection times in case of some participants due to their limited availability in their offices. We suggest future studies interested in studying employee experiences at work in different work settings employ ESM design that does not require employees to be physically present at their desk and that allows responding (e.g., via smartphone) from any location at work (e.g., meeting rooms). Also, due to constraints in the supervisors’ availability, we obtained supervisor ratings of their employees’ extra-role performance only once, at the end of the measurement week. Therefore, we could not analyze the daily co-fluctuations in state eudaimonic well-being and state extra-role performance assessed by the supervisor, taking into account the time of the day (morning vs. afternoon). Future research should consider incorporating more frequent supervisor assessments of their employees’ extra-role performance. Fourth, in this study we used i*SD* to quantify intra-individual variability, a measure that has been the most extensively used index of variability across time, is familiar to many researchers [120] and face valid [84]. Although i*SD* does not allow to capture the temporal sequence of variations as opposed to other quantifications (e.g., *MSSD*) [120], the main focus of our study is the amplitude of fluctuations quantified by the i*SD* [121]. Although i*SD* is less sensitive to systematic intra-individual long-term change over time as compared to *MSSD* [120], this is not an issue in case of short-term fluctuations in this study. Also, i*SD* and *MSSD* indexes are highly related [120]. Fifth, a potential limitation of our research might be a social desirability bias to self-report data about ones’ performance. To minimize it, the scale instructions in this study emphasized that all answers are completely confidential and that there are no right or wrong answers. Finally, given the complexity of our models and the number of parameters estimated, we used a first-order BSEM instead of a second-order BSEM. A critical contribution of second-order models, especially relevant for repeated-measures data, is that they make it possible to test longitudinal factorial invariance [113]. However, we were able to test it using an additional DIF analysis, which indicated strong factorial invariance, providing support for the assumption that the variables used in our study retain the same meaning across all eight data collection points.

The present study contributes to unfolding the complex dynamic relationship between the eudaimonic facet of well-being and extra-role performance, which had not been sufficiently explored [6]. This dynamic perspective on the relationship between eudaimonic well-being and extra-role performance sheds light on how to enhance the sustainability of both well-being and performance over time [1]. Only by combining these two will it be possible to achieve a fair exchange between workers and their organizations. Indeed, intrinsic motivation, fulfilling the mission at work, or self-realization cannot be attained by considering only the hedonic aspect of well-being [20]. Moreover, knowledge about the dynamic outcomes of the variability in the eudaimonic component of well-being [6,43] enriches the happy-productive worker model, which has produced inconclusive results so far, possibly due to neglecting the impact of within-person fluctuations in well-being on performance [21]. Furthermore, the study follows the advances in the measurement of subjective well-being [47] by distinguishing the worthwhileness aspect of the activities carried out every day [13,14]. The adoption of this micro daily and weekly temporal lens on four consecutive workdays through a diary study design makes it possible to show the complex nature of the underlying processes in the dynamic relationships between eudaimonic well-being and extra-role performance [6]. This is not possible when merely assuming that well-being and performance are global experiences [54] measured with questionnaires that capture their overall stable evaluations. Finally, in this study we combine employees’ ratings of their state extra-role performance with the examination of overall levels of employees’ extra-role performance as assessed by their direct supervisors, in order to avoid employees’ leniency or self-deception in self-ratings, which tends to occur in cases of general or trait judgments of performance [85].

The study has practical implications stemming from the knowledge acquired. First, supervisors and companies should keep in mind that eudaimonic well-being is a state-like experience that presents fluctuations over time. These fluctuations are important per se and ensure the sustainability of well-being and the dynamics of performance. Second, this study draws our attention to the fact that it is not sufficient to focus exclusively on enhancing hedonic well-being at work (e.g., by promoting positive emotions, comfort, satisfaction). Indeed, the daily experience of meaning at work should complement the experience of hedonic well-being because it is an important factor that boosts individual extra-role performance on a daily basis. Finally, this study suggests that saturating our working day with meaning (e.g., through task planning or coaching) is important in itself, and a few highly meaningful activities at work should not just be sparsely distributed to compensate for jobs full of insignificant tasks.

## Supporting information

S1 Table Descriptive statistics and correlations for hedonic well-being, eudaimonic well-being, and extra-role performance. (DOCX)

S1 Dataset Dataset file. (XLSX)
