# Supplementary material for: Daily eudaimonic well-being as a predictor of daily performance: A dynamic lens
Source: PLoS One. 2019 Apr 19;14(4):e0215564. doi: 10.1371/journal.pone.0215564 (PMC6474601; doi:10.1371/journal.pone.0215564)
Supplement: S1 Table — (DOCX) [file pone.0215564.s001.docx]

S1 Table.

*Descriptive statistics and correlations for hedonic well-being, eudaimonic well-being, and extra-role performance*

|  |  | *M* | *SD* | *N* | *1* | *2* | *3* | *4* | *5* | *6* |
| --- | --- | --- | --- | --- | --- | --- | --- | --- | --- | --- |
| 1 | variability in hedonic well-being | 0.79 | 0.37 | 83 |  |  |  |  |  |  |
| 2 | hedonic well-being t1 | 4.65 | 1.14 | 79 | -.24^**^ | (.77) |  |  |  |  |
| 3 | hedonic well-being t2 | 4.71 | 1.08 | 73 | -.15 | .74^**^ | (.77) |  |  |  |
| 4 | hedonic well-being t3 | 4.71 | 1.16 | 80 | -.28^**^ | .67^**^ | .75^**^ | (.72) |  |  |
| 5 | hedonic well-being t4 | 4.62 | 1.24 | 78 | -.40^**^ | .63^**^ | .67^**^ | .81^**^ | (.68) |  |
| 6 | hedonic well-being t5 | 4.72 | 1.25 | 76 | -.35^**^ | .70^**^ | .73^**^ | .78^**^ | .81^**^ | (.72) |
| 7 | hedonic well-being t6 | 4.73 | 1.27 | 69 | -.41^**^ | .71^**^ | .59^**^ | .68^**^ | .70^**^ | .77^**^ |
| 8 | hedonic well-being t7 | 4.84 | 1.25 | 74 | -.21 | .66^**^ | .72^**^ | .79^**^ | .72^**^ | .72^**^ |
| 9 | hedonic well-being t8 | 4.72 | 1.29 | 74 | -.42^**^ | .75^**^ | .61^**^ | .66^**^ | .69^**^ | .71^**^ |
| 10 | variability in eudaimonic well-being | 0.66 | 0.44 | 83 | .24^**^ | -.22^**^ | -.25^**^ | -.27^**^ | -.27^**^ | -.18 |
| 11 | eudaimonic well-being t1 | 5.03 | 1.25 | 79 | -.05 | .47^**^ | .42^**^ | .45^**^ | .40^**^ | .36^**^ |
| 12 | eudaimonic well-being t2 | 5.21 | 1.07 | 73 | .06 | .33^**^ | .49^**^ | .37^**^ | .29^**^ | .25^**^ |
| 13 | eudaimonic well-being t3 | 5.03 | 1.27 | 80 | -.10 | .43^**^ | .35^**^ | .39^**^ | .44^**^ | .36^**^ |
| 14 | eudaimonic well-being t4 | 5.14 | 1.27 | 78 | -.14 | .44^**^ | .36^**^ | .41^**^ | .45^**^ | .38^**^ |
| 15 | eudaimonic well-being t5 | 5.26 | 1.18 | 76 | -.15 | .48^**^ | .47^**^ | .44^**^ | .44^**^ | .46^**^ |
| 16 | eudaimonic well-being t6 | 5.29 | 1.35 | 69 | -.19 | .50^**^ | .36^**^ | .40^**^ | .44^**^ | .39^**^ |
| 17 | eudaimonic well-being t7 | 5.31 | 1.19 | 74 | -.17 | .51^**^ | .54^**^ | .46^**^ | .51^**^ | .49^**^ |
| 18 | eudaimonic well-being t8 | 5.23 | 1.33 | 74 | -.25^**^ | .56^**^ | .49^**^ | .44^**^ | .46^**^ | .52^**^ |
| 19 | overall extra-role performance^1^ | 5.45 | 1.36 | 75 | .08 | .14 | .10 | -.03 | .11 | .12 |
| 20 | extra-role performance t1 | 4.23 | 1.52 | 79 | -.15 | .27^**^ | .39^**^ | .28^**^ | .39^**^ | .28^**^ |
| 21 | extra-role performance t2 | 4.16 | 1.49 | 73 | -.01 | .19 | .34^**^ | .21 | .26^**^ | .18 |
| 22 | extra-role performance t3 | 4.04 | 1.50 | 80 | -.16 | .21 | .38^**^ | .31^**^ | .38^**^ | .31^**^ |
| 23 | extra-role performance t4 | 4.31 | 1.54 | 78 | -.18 | .19 | .27^**^ | .14 | .33^**^ | .26^**^ |
| 24 | extra-role performance t5 | 4.19 | 1.49 | 76 | -.05 | .24^**^ | .32^**^ | .13 | .36^**^ | .29^**^ |
| 25 | extra-role performance t6 | 4.32 | 1.52 | 69 | -.09 | .33^**^ | .37^**^ | .18 | .38^**^ | .28^**^ |
| 26 | extra-role performance t7 | 4.13 | 1.42 | 74 | -.19 | .27^**^ | .43^**^ | .30^**^ | .45^**^ | .37^**^ |
| 27 | extra-role performance t8 | 4.00 | 1.50 | 74 | -.21 | .33^**^ | .49^**^ | .40^**^ | .50^**^ | .38^**^ |

***Note.*** ^1^evaluated by the direct supervisor; ^**^ correlation is significant at .01 level; The numbers in brackets represent the Cronbach alphas for the scales.

S1 Table (continued)

*Descriptive Statistics and Correlations for Hedonic Well-Being, Eudaimonic Well-Being, and Extra-Role Performance*

|  |  | *7* | *8* | *9* | *10* | *11* | *12* | *13* | *14* | *15* | *16* |
| --- | --- | --- | --- | --- | --- | --- | --- | --- | --- | --- | --- |
| 7 | hedonic well-being t6 | (.76) |  |  |  |  |  |  |  |  |  |
| 8 | hedonic well-being t7 | .75^**^ | (.73) |  |  |  |  |  |  |  |  |
| 9 | hedonic well-being t8 | .80^**^ | .77^**^ | (.69) |  |  |  |  |  |  |  |
| 10 | variability in eudaimonic well-being | -.19 | -.14 | -.28^**^ |  |  |  |  |  |  |  |
| 11 | eudaimonic well-being t1 | .27^**^ | .53^**^ | .46^**^ | -.31^**^ | (.82) |  |  |  |  |  |
| 12 | eudaimonic well-being t2 | .22 | .46^**^ | .25^**^ | -.19 | .83^**^ | (.79) |  |  |  |  |
| 13 | eudaimonic well-being t3 | .27^**^ | .48^**^ | .46^**^ | -.41^**^ | .73^**^ | .68^**^ | (.79) |  |  |  |
| 14 | eudaimonic well-being t4 | .35^**^ | .49^**^ | .51^**^ | -.38^**^ | .74^**^ | .68^**^ | .83^**^ | (.75) |  |  |
| 15 | eudaimonic well-being t5 | .50^**^ | .54^**^ | .61^**^ | -.28^**^ | .72^**^ | .66^**^ | .73^**^ | .84^**^ | (.80) |  |
| 16 | eudaimonic well-being t6 | .50^**^ | .56^**^ | .55^**^ | -.20 | .66^**^ | .66^**^ | .68^**^ | .79^**^ | .88^**^ | (.82) |
| 17 | eudaimonic well-being t7 | .44^**^ | .55^**^ | .60^**^ | -.42^**^ | .73^**^ | .71^**^ | .84^**^ | .84^**^ | .83^**^ | .79^**^ |
| 18 | eudaimonic well-being t8 | .49^**^ | .55^**^ | .63^**^ | -.49^**^ | .60^**^ | .53^**^ | .73^**^ | .77^**^ | .81^**^ | .82^**^ |
| 19 | overall extra-role performance^1^ | .07 | .03 | .23 | .01 | -.02 | -.10 | .12 | .12 | .00 | -.06 |
| 20 | extra-role performance t1 | .32^**^ | .32^**^ | .22 | -.29^**^ | .36^**^ | .34^**^ | .34^**^ | .49^**^ | .33^**^ | .42^**^ |
| 21 | extra-role performance t2 | .20 | .24^**^ | .04 | -.28^**^ | .33^**^ | .39^**^ | .28^**^ | .42^**^ | .27^**^ | .37^**^ |
| 22 | extra-role performance t3 | .22 | .30^**^ | .11 | -.31^**^ | .30^**^ | .30^**^ | .37^**^ | .41^**^ | .32^**^ | .33^**^ |
| 23 | extra-role performance t4 | .21 | .21 | .14 | -.28^**^ | .28^**^ | .30^**^ | .36^**^ | .44^**^ | .33^**^ | .40^**^ |
| 24 | extra-role performance t5 | .23 | .23 | .17 | -.18 | .28^**^ | .35^**^ | .24^**^ | .37^**^ | .39^**^ | .47^**^ |
| 25 | extra-role performance t6 | .22 | .33^**^ | .30^**^ | -.13 | .32^**^ | .35^**^ | .33^**^ | .45^**^ | .44^**^ | .50^**^ |
| 26 | extra-role performance t7 | .16 | .32^**^ | .27^**^ | -.27^**^ | .29^**^ | .29^**^ | .25^**^ | .38^**^ | .32^**^ | .30^**^ |
| 27 | extra-role performance t8 | .31^**^ | .44^**^ | .32^**^ | -.32^**^ | .30^**^ | .28^**^ | .28^**^ | .36^**^ | .31^**^ | .38^**^ |

***Note.*** ^1^evaluated by the direct supervisor; ^**^correlation is significant at .01 level; The numbers in brackets represent the Cronbach alphas for the scales.

S1 Table (continued)

*Descriptive Statistics and Correlations for Hedonic Well-Being, Eudaimonic Well-Being, and Extra-Role Performance*

|  |  | *17* | *18* | *19* | *20* | *21* | *22* | *23* | *24* | *25* | *26* | *27* |
| --- | --- | --- | --- | --- | --- | --- | --- | --- | --- | --- | --- | --- |
| 17 | eudaimonic well-being t7 | (.79) |  |  |  |  |  |  |  |  |  |  |
| 18 | eudaimonic well-being t8 | .94^**^ | (.83) |  |  |  |  |  |  |  |  |  |
| 19 | overall extra-role performance^1^ | .15 | .12 | (.86) |  |  |  |  |  |  |  |  |
| 20 | extra-role performance t1 | .33^**^ | .31^**^ | .20 | (.74) |  |  |  |  |  |  |  |
| 21 | extra-role performance t2 | .31^**^ | .26^**^ | .18 | .82^**^ | (.76) |  |  |  |  |  |  |
| 22 | extra-role performance t3 | .39^**^ | .26^**^ | .15 | .77^**^ | .77^**^ | (.74) |  |  |  |  |  |
| 23 | extra-role performance t4 | .38^**^ | .41^**^ | .20 | .70^**^ | .71^**^ | .80^**^ | (.75) |  |  |  |  |
| 24 | extra-role performance t5 | .32^**^ | .39^**^ | .08 | .67^**^ | .75^**^ | .65^**^ | .78^**^ | (.76) |  |  |  |
| 25 | extra-role performance t6 | .40^**^ | .47^**^ | .12 | .63^**^ | .68^**^ | .64^**^ | .77^**^ | .84^**^ | (.73) |  |  |
| 26 | extra-role performance t7 | .44^**^ | .42^**^ | .16 | .54^**^ | .59^**^ | .67^**^ | .67^**^ | .72^**^ | .81^**^ | (.69) |  |
| 27 | extra-role performance t8 | .43^**^ | .47^**^ | .12 | .65^**^ | .67^**^ | .68^**^ | .67^**^ | .67^**^ | .80^**^ | .86^**^ | (.72) |

***Note.*** ^1^evaluated by the direct supervisor; ^**^correlation is significant at .01 level; The numbers in brackets represent the Cronbach alphas for the scales.
